# Clinical Application of High-Frame-Rate Vector Flow Imaging in Evaluation of Carotid Atherosclerotic Stenosis

**DOI:** 10.3390/diagnostics13030519

**Published:** 2023-01-31

**Authors:** Yi-Jie Qiu, Juan Cheng, Qi Zhang, Dao-Hui Yang, Dan Zuo, Feng Mao, Ling-Xiao Liu, Yi Dong, Si-Qi Cao, Wen-Ping Wang

**Affiliations:** 1 Department of Ultrasound, Zhongshan Hospital, Fudan University, Shanghai 200032, China; 2 Department of Ultrasound, Xinhua Hospital Affiliated to Shanghai Jiaotong University School of Medicine, Shanghai 200092, China; 3 Department of Interventional Radiology, Zhongshan Hospital, Fudan University, Shanghai 200032, China

**Keywords:** vector flow (V flow), high frame rate, carotid atherosclerotic stenosis, wall shear stress (WSS), ultrasound

## Abstract

Objective: This study seeks to evaluate the value of the high-frame-rate vector flow imaging technique in assessing the hemodynamic changes of carotid atherosclerotic stenosis in aging people (>60 years old). Methods: Aging patients diagnosed with carotid atherosclerotic stenosis who underwent carotid high-frame-rate vector flow imaging examination were prospectively enrolled. A Mindray Resona7s ultrasound machine equipped with high-frame-rate vector flow function was used for ultrasound evaluation. First, B mode ultrasound and color Doppler flow imaging were used to evaluate carotid stenosis. Then, the vector arrows and flow streamline detected by V Flow were analyzed and the wall shear stress values (Pa) at the carotid stenosis site were measured. All patients were divided into symptomatic and asymptomatic groups according to whether they had acute/subacute stroke or other clinical symptoms within 2 weeks before ultrasound examination. The results of digital subtraction angiography or computed tomography angiography were used as the gold standard. The stenosis rate was calcified, according to North American Symptomatic Carotid Endarterectomy Trial criteria. The diagnostic values of wall shear stress, conventional ultrasound, and the combined diagnosis in carotid atherosclerotic stenosis were compared. Results: Finally, 88 patients with carotid atherosclerotic plaque were enrolled (71 males (80.7%), mean age 67.6 ± 5.4 years). The success rate of high-frame-rate vector flow imaging was 96.7% (88/91). The WSS value of symptomatic carotid stenosis (1.4 ± 0.15 Pa) was significantly higher than that of asymptomatic carotid stenosis (0.80 ± 0.08 Pa) (*p* < 0.05). Taking the wall shear stress value > 0.78 Pa as the diagnostic criteria for symptomatic carotid atherosclerotic plaque, the area under receiver operating characteristic curves was 0.79 with 87.1% sensitivity and 69.6% specificity. The area under receiver operating characteristic curves of the combined diagnosis (0.966) for differentiating severe carotid atherosclerotic stenosis was significantly higher than that of conventional ultrasound and WSS value, with 89.7% sensitivity and 93.2% specificity (*p* < 0.05). Conclusion: As a non-invasive imaging method, the high-frame-rate vector flow imaging technique showed potential value in the preoperative assessment of the symptomatic carotid atherosclerotic stenosis and diagnosing carotid atherosclerotic stenosis in aging patients.

## 1. Introduction

Carotid atherosclerotic stenosis is the main cause of ischemic stroke, which may cause long-term disability and mortality in aging people [1,2]. It has been reported that moderate and severe (50–99%) carotid stenosis might affect 9% of the general population of aging people (>60 years old) and cause 10% of all strokes [3,4,5]. The medical burden of patients with carotid stenosis is severe. Symptomatic carotid stenosis refers to patients with previous stroke or transient ischemic attack (TIA), or a combination of these [4]. The imaging screening and follow-up of symptomatic carotid stenosis is of great significance to the prognosis and quality of life of the aging patients. According to European Society for Vascular Surgery guidelines, current clinical indication for surgery in carotid stenosis patients includes ≥70% carotid stenosis in symptomatic patients on non-invasive imaging modalities or >50% stenosis on DSA [6]. The identification of the stenosis rate is of great significance in the clinical decision-making process. Magnetic resonance imaging (MRI) could assess the property and morphology of the atherosclerotic plaque, with typical features including the lipid core and intraplaque hemorrhage [7]. However, the longer scan time, higher cost, and metal contraindications were the main disadvantages of MRI [7,8]. Computed tomography angiography (CTA) showed the potential in clinical diagnosis and fluid dynamics of carotid plaques [9,10]. The potential exposure to radiation limited its clinical applications. Ultrasound is the first-line medical imaging modality for the assessment of the hemodynamics of carotid stenosis. The common ultrasound manifestations of carotid atherosclerotic stenosis are the increase in carotid intima media thickness, atherosclerotic plaque formation, local lumen stenosis, and increased peak systolic velocity at the stenosis site [11]. Despite the limitations of ultrasound in diagnosis of carotid stenosis such as calcification or the subjectivity of the observer, previous studies have shown that the sensitivity and specificity were 82% and 76% in diagnosing severe carotid stenosis, and 34% and 85% in diagnosing moderate carotid stenosis by conventional ultrasound [12]. Accurate diagnosis of carotid stenosis makes a difference to the patients’ treatment decisions [13]. The diagnostic value of different degrees of carotid stenosis still needs to be further improved.

The high-frame-rate vector flow imaging technique (V Flow) is a novel ultrasound technique that can facilitate the assessment of carotid arteries using the intuitive and quantitative vector arrows [14,15]. It can depict the blood flow features of carotid arteries in a real-time scan, especially the complex blood flow [16]. In addition, V Flow can provide the quantitative parameter named wall shear stress (WSS), which is the frictional force between blood flow and the endothelial surface of the vessel wall [17,18,19]. Non-invasive measurement of WSS may provide more information in the diagnosis and decision-making process of carotid stenosis [20]. In our previous work, V Flow proved to be a feasible imaging method for the WSS assessment of the common carotid artery in healthy volunteers [18]. However, the value of V Flow for assessing the carotid stenosis in aging patients has not been reported.

The aim of this study was to explore the value of V Flow in assessing symptomatic carotid atherosclerotic stenosis and evaluating the degree of carotid stenosis in aging people (>60 years old).

## 2. Methods

### 2.1. Patients

This prospective study was approved by the Ethics Committee of Zhongshan Hospital, Fudan University (no. B2019-295R), and informed consent was obtained in all patients. The inclusion criteria were as follows: (1) patients older than 60 years old (aging patients); (2) patients with carotid stenosis caused by carotid atherosclerotic plaques; (3) patients who underwent V Flow imaging examination before surgery; (4) digital subtraction angiography (DSA) or CTA examination was the gold standard for the carotid stenosis. Exclusion criteria included (1) patients’ carotid could not be observed clearly on B mode ultrasound (BMUS); (2) DSA or CTA indicated complete carotid occlusion.

All patients were divided into symptomatic and asymptomatic groups according to whether they had clinical symptoms within 2 weeks before the ultrasound examination and/or whether their head MRI showed acute/subacute stroke [7,21]. Clinical symptoms include classic TIA and anterior circulation ischemic stroke [22], as well as monocular symptoms (amaurosis fugax or retinal artery occlusion) ipsilateral to the carotid atherosclerotic plaque [7,23]. Baseline characteristics of patients, including sex, age, and risk factors for atherosclerotic plaque (hypertension, diabetes, and hyperlipidemia) were recorded.

### 2.2. Ultrasound Examination

The examination was performed using Mindray Resona7s (Shenzhen Mindray Bio-Medical Electronic Co., Shenzhen, China) with a L9-3 linear array probe. The ultrasound machine was equipped with a novel V Flow function for carotid vector flow imaging. The patients laid in a supine position with a thin pillow behind the neck. First transversal and longitudinal BMUS scans were performed on the common carotid artery, internal carotid artery, and external carotid artery. The carotid atherosclerotic plaque was detected and measured. For carotid stenosis, the echogenicity of atherosclerotic plaques, the residual lumen diameter, and distal normal lumen diameter of the carotid artery stenosis were observed. The peak systolic velocity (PSV), end-diastolic velocity (EDV), and resistance index (RI) at the carotid stenosis site and common carotid artery were measured by color Doppler flow imaging (CDFI). The carotid artery stenosis rate can be calculated by the following: Stenosis rate = (1 − residual lumen diameter at the stenosis/normal lumen diameter at the distal of the stenosis) × 100% [24]. The stenosis degrees of the carotid artery were stratified into the categories of mild (<50%), moderate (50–69%), or severe (70–99%) degrees according to North American Symptomatic Carotid Endarterectomy Tria (NASCET) criteria [24,25]. The diagnosis of carotid stenosis was in accordance with the Society of Radiologists in Ultrasound Consensus Conference for carotid artery stenosis by gray-scale and Doppler US [25].

After the carotid artery stenosis was clearly detected on BMUS, the “V Flow” mode was switched to. Then, the “Update” button was pressed to acquire the dynamic V Flow image. The V Flow scanning settings were as follows: the frequency was 5.0 MHz, the depth was 2–4 cm, the V flow arrow density was 10%, and the arrow life cycle was 25 ms. The direction, color, and streamline of vector arrows were observed and recorded. The vector arrows were defined as follows: the redder vector arrow was associated with the faster blood flow; the greener vector arrow was associated with the slower blood flow; the longer vector arrow was associated with the faster blood flow; and the direction of the vector arrow signifies the direction of blood flow. In addition, flow streamlines represent the overall flow of blood. Then, the WSS value during the systolic phase of the cardiac cycle at the carotid stenosis site was measured. The WSS value on the vessel wall as a function of time was estimated on ultrasound machine by the following:

(1) $$\underset{\tau }{\to }\left(t\right)=\frac{1}{N}\mu \overset{i=N}{\underset{i=1}{\sum }}\frac{\underset{w}{\to }.\;\underset{v_{i}}{\to }\left(t\right)}{\Delta r_{i}}$$

where *μ* is blood viscosity; $\underset{w}{\to }$ denotes the direction of $\;\underset{\tau }{\to }$ and is a unit vector; $\;\underset{v_{i}}{\to }$ is the vector velocity; and $\Delta r_{i}$ is the distance between the ith velocity measurement and the WSS measurement location [20].

All ultrasound examinations were performed by a radiologist with > 5 years’ experience in carotid ultrasound examination.

### 2.3. Gold Standard

DSA or CTA examinations were performed as gold standard for the patients with carotid stenosis. The inner diameter of the distal normal lumen was taken as the reference inner diameter (A), and the inner diameter of the residual lumen at the stenosis segment was taken as the measurement value (B). The degree of carotid stenosis was (1 − B/A) × 100% according to NASCET criteria [24].

### 2.4. Statistical Analysis

The independent samples *t*-test was used to compare the WSS values of carotid atherosclerotic stenosis. Correlation analysis was performed using Pearson’s correlation coefficient. Receiver operating characteristic curves (ROC) was used to compare the efficacy of WSS values in assessing the symptomatic carotid stenosis. Comparison of the diagnostic value of WSS, conventional ultrasound, and the combined diagnosis in carotid atherosclerotic stenosis was analyzed. All data were calculated using the software program GraphPad prism 7 (GraphPad Software, Inc., Boston, MA, USA) and MedCalc (MedCalc Software, Ltd., Ostend, Belgium); *p* < 0.05 was statistically significant.

## 3. Results

### 3.1. General Characteristics

Finally, 88 patients (mean age 67.6 ± 5.4 years) with carotid atherosclerotic plaque were enrolled, including 71 males (80.7%) and 17 females (19.3%). Hypertension, diabetes, and hyperlipidemia were identified in 47.7%, 10.2%, and 19.3% of patients as the potential risk factors for carotid atherosclerotic plaques. All patients were divided into the symptomatic group (32, 36.4%) and asymptomatic group (56, 63.6%). According to DSA or CTA results, there were 44 cases of mild carotid stenosis, 15 moderate carotid stenosis, and 29 severe carotid stenosis (Table 1).

### 3.2. Conventional Ultrasound Imaging Features

Among all carotid plaques, 37 (42.0%) were hypoechoic, 27 (30.7%) were mixed echoic, 18 (20.5%) were hyperechoic, and 6 (6.8%) were isoechoic on BMUS. Meanwhile, 37 mild stenosis cases, 26 moderate stenosis, and 25 severe stenosis were diagnosed on BMUS. The mean PSV measured at the site of carotid stenosis was 164.25 ± 104.42 cm/s, and the RI value at the carotid stenosis site was 0.70 ± 0.10.

### 3.3. V Flow Imaging Features

The success rate of V Flow imaging was 96.7% (88/91), including 98.3% (59/60) of patients with mild to moderate carotid stenosis and 93.5% (29/31) of patients with severe stenosis. These three patients with failed V Flow dynamic imaging were excluded. The reason for the failed V Flow dynamic imaging in three patients was the occlusion of acoustic shadows caused by large calcifications in the carotid plaques, which affected the effect of vector flow dynamic imaging.

**Figure 1:**
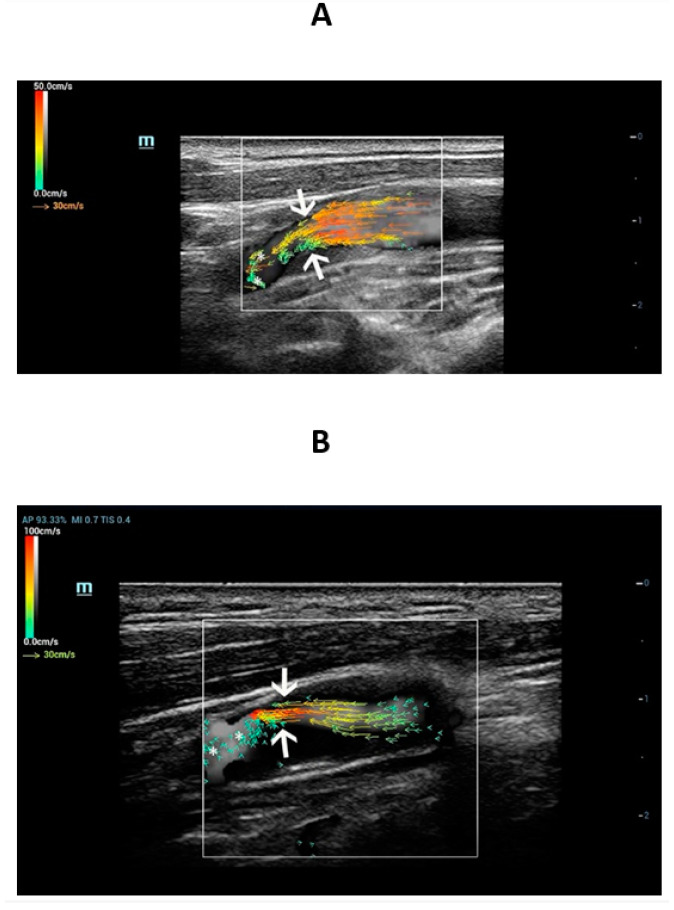
High-frame-rate vector flow imaging of the carotid stenosis. Moderate stenosis of the right internal carotid artery (61, male) (**A**) and severe stenosis of the left internal carotid artery (67, male) (**B**). The vector arrows at the stenosis (arrow) site became longer and the color of the vector arrow was red. V Flow could intuitively show changes of flow velocity at the carotid stenosis site. Complex blood flow such as vortex (short green or yellow vectors) (asterisk) could be detected at the distal area of the stenosis.

The V Flow dynamic imaging shows that the length of the vector arrows at site of the carotid stenosis was longer than the surrounding vessel wall (Figure 1). The color of vector arrows at the carotid stenosis site was orange or red with faster flow velocity (Figure 1). However, the most common vector arrows at the distal and proximal sites of the stenosis were slower green or yellow vector arrow (Figure 1).

The V Flow streamline showed that there was complex blood flow such as reflux and vortex at the distal area of carotid stenosis, which were represented by reversed or spiral vector arrows. V Flow could provide blood flow visualization of the complex blood flow at the distal area of the carotid stenosis (Figure 1).

### 3.4. Diagnostic Value of WSS Value in Distinguishing Symptomatic Carotid Stenosis

**Figure 2:**
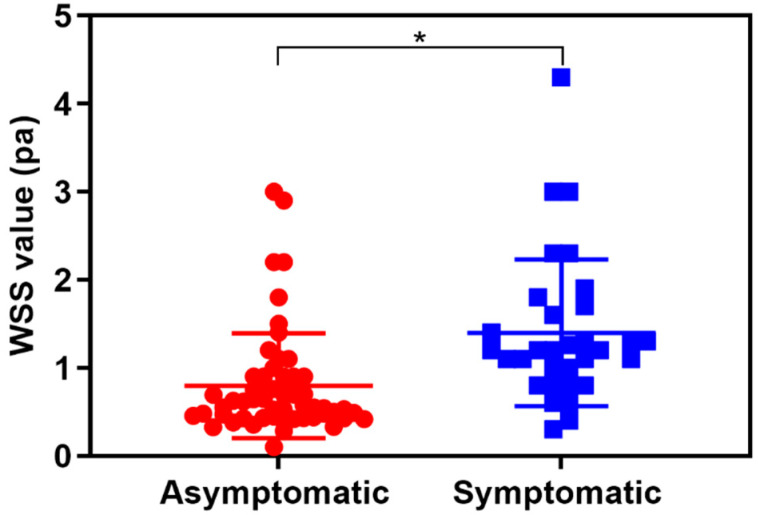
The wall shear stress (WSS) value of the asymptomatic and symptomatic carotid stenosis. * *p* < 0.05.

**Figure 3:**
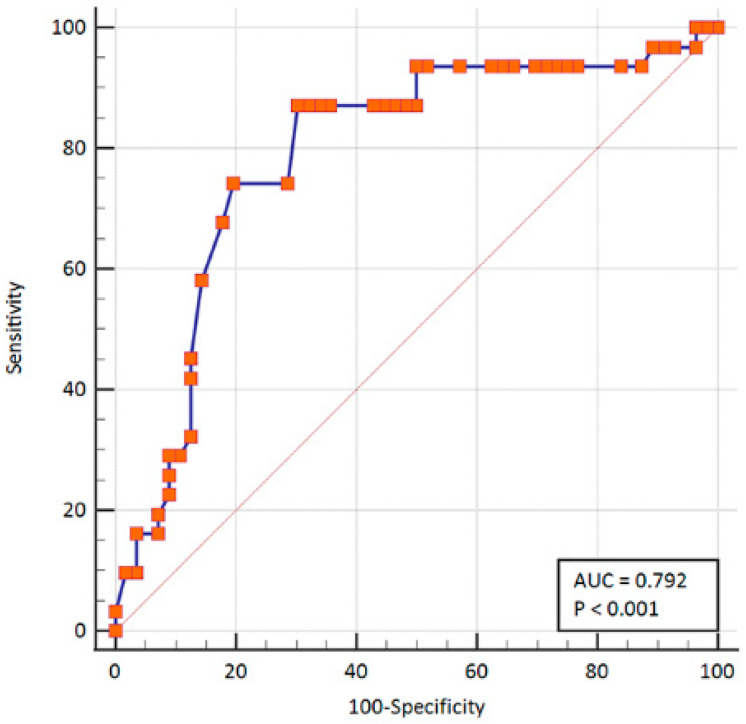
The diagnostic value of wall shear stress (WSS) in distinguishing between the asymptomatic and symptomatic carotid atherosclerotic stenosis.

The WSS value of symptomatic carotid stenosis (1.4 ± 0.15 Pa) measured at the stenotic segment was significantly higher than that of asymptomatic carotid stenosis (0.80 ± 0.08 Pa) (*p* < 0.05) (Figure 2). Taking a WSS value > 0.78 Pa as diagnostic criteria for symptomatic carotid atherosclerotic stenosis, the AUROC was 0.79 with 87.1% sensitivity and 69.6% specificity (Figure 3). Furthermore, significant differences in WSS values of symptomatic and asymptomatic patients were independent of the carotid stenosis grade (*p* < 0.05).

### 3.5. Comparison of Diagnostic Efficacy of WSS Value and Conventional Ultrasound in Carotid Atherosclerotic Stenosis

The mean WSS value of the stenotic segment in mild, moderate, and severe carotid stenosis was 0.53 ± 0.14 Pa, 0.99 ± 0.51 Pa, and 1.66 ± 0.87 Pa, respectively (*p* < 0.05). The Pearson correlation between the mean WSS value and the degree of the carotid stenosis was 0.65 (*p* < 0.05).

**Figure 4:**
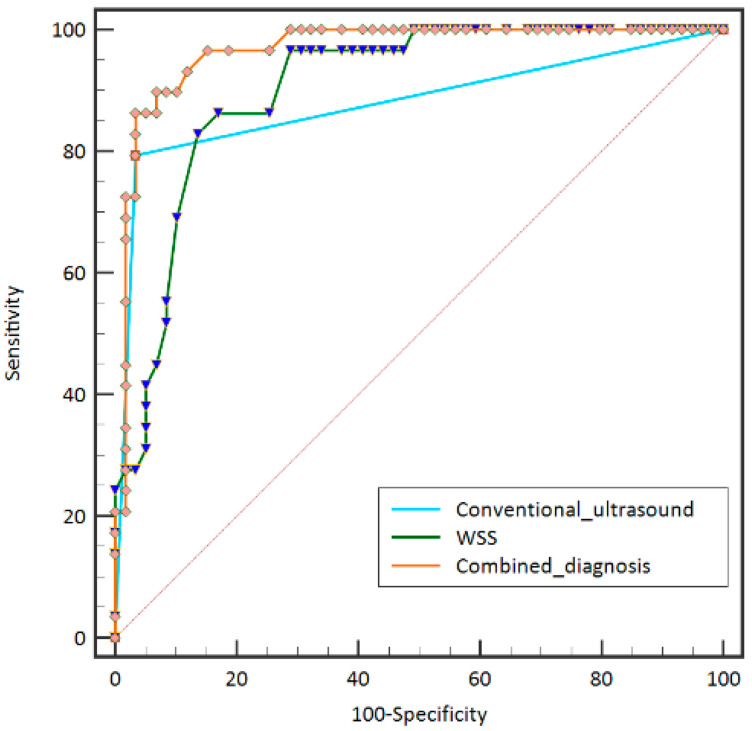
The comparison of diagnostic efficacy of wall shear stress (WSS), conventional ultrasound, and combined diagnosis in differentiating the severe carotid atherosclerotic stenosis.

With WSS value > 0.90 Pa as the cut-off value, the AUROC for severe carotid atherosclerotic stenosis was 0.90 with 86.2% sensitivity and 83.1% specificity. For conventional ultrasound, the sensitivity and specificity for severe carotid atherosclerotic stenosis were 79.3% and 96.6%, respectively (Table 2). No significant difference was detected between the WSS value and the conventional ultrasound for diagnosing severe carotid stenosis (*p* > 0.05). However, the AUROC of the combined diagnosis (0.966) was significantly higher than that of the conventional ultrasound and WSS value with 89.7% sensitivity and 93.2% specificity (*p* < 0.05) (Figure 4).

## 4. Discussion

Aging was reported as one of the main risk factors related to carotid atherosclerotic stenosis, and the elder populations > 60 y are the high-risk group of carotid stenosis [26]. Individuals with carotid atherosclerosis are at an increasing risk of future cardiovascular disease, such as myocardial infarction (heart attack), ischemic stroke, and peripheral arterial disease [27]. Comparing to conventional imaging methods, V Flow is a brand new vector flow imaging technology which could provide various additional quantitative and intuitive parameters for the evaluating the hemodynamics of the carotid artery [15]. WSS is the frictional force between blood flow and the endothelial surface of the vessel wall [17,18]. The local hemodynamic factors such as WSS influence the initiation and progression of atherosclerosis plaques [28]. It is well known that low and oscillatory WSS values are associated with thickening of the arterial intima and the development of atherosclerosis [29]. Meanwhile, the high WSS has been reported to be closely associated with coronary plaque rupture [28,30,31]. The V Flow technique is convenient to measure the WSS value at the site of carotid stenosis. For patients with a high WSS value, carotid endarterectomy should be recommended to avoid plaque shedding during surgery. The preoperative identification of the vulnerable plaque is critical to the surgical option. However, the relationship between the WSS values detected by V Flow and carotid plaque vulnerability has not been elucidated. Our results showed that the WSS value of symptomatic carotid stenosis was significantly higher than that of asymptomatic carotid stenosis (*p* < 0.05). Patients with higher WSS values have poor plaque stability, and carotid endarterectomy should be performed to avoid intraoperative plaque shedding. Tuenter et al. also reported that higher maximum WSS detected by MRI was associated with vulnerable plaque components [32], which is consistent with our results. Then, we further investigated the efficacy of WSS values in diagnosing symptomatic carotid atherosclerotic stenosis. Taking a WSS value > 0.78 Pa as the diagnostic criteria for symptomatic carotid stenosis, the AUROC for diagnosing symptomatic carotid atherosclerotic stenosis was 0.79 with 87.1% sensitivity and 69.6% specificity. The WSS value of the carotid stenosis measured by V Flow may be a potential indicator for predicting vulnerable plaque and plaque rupture.

V Flow measures the axial and transverse vector velocity and direction of the blood flow, providing both spatial and temporal vector information without the need for angle correction [16]. A previous study has proved that the V Flow measures the flow velocity more accurately than CDFI [33]. Meanwhile, the vector arrows at the stenosis site became longer, and the color of the vector arrow was red in our research. The color and length of vector arrows provide visual quantification of the blood flow. Vector flow mapping technology shows promising results in the quantitative assessment of differences in the hemodynamics of the vascular flow field between patients with hypertension and normal controls [34]. Complex blood flow such as vortex (short green or yellow vectors) could be visually detected at the distal region of the stenosis by V Flow in our study. CDFI has also been used to detect blood flow disturbances at carotid bifurcations through assessing blood flow patterns [35]. However, CDFI has a limited frame rate and cannot quantify complex blood flow. These limit CDFI in assessing complex blood flow for clinical diagnosis [16]. Previous studies reported that V Flow was more powerful in the assessment of complex flow patterns than CDFI [14]. By considering the direction, length, and streamline of the vector arrows in V Flow, flow characteristics of the carotid stenosis can be evaluated visually for assessing flow patterns.

In our results, the WSS value (1.66 ± 0.87 Pa) at the carotid stenosis site in patients with severe carotid stenosis was significantly higher than those with moderate carotid stenosis (0.99 ± 0.51 Pa) and mild carotid stenosis (0.53 ± 0.14 Pa) (*p* < 0.05). The WSS value was positively correlated with carotid stenosis rate (*p* < 0.05). According to the theoretical studies, the WSS value was positively correlated with carotid blood flow velocity [20]. These results indicated the accuracy and feasibility of V Flow to measure WSS at the carotid stenosis site. Higher WSS values could be observed in severe stenosis, which promotes complex flow or an increased flow velocity [20]. The carotid artery hemodynamic changes detected by CDFI is the routine approach for assessing carotid stenosis. The reported sensitivity was 82% and the specificity was 76% for distinguishing severe carotid stenosis by conventional ultrasound [12]. In our study, there was similar sensitivity and higher specificity for diagnosing severe carotid stenosis with conventional ultrasound. Taking the WSS value of the stenosis greater than 0.90 Pa as the cut-off value for severe carotid stenosis, no significant difference was detected in the performance of the two techniques in diagnosing severe carotid stenosis (*p* > 0.05). However, when the combined diagnosis of WSS value and conventional ultrasound was performed, a significantly higher AUROC was obtained with 89.7% sensitivity and 93.2% specificity (*p* < 0.05). The combined use of WSS and conventional ultrasound has broad application prospects in the diagnosis of severe carotid stenosis. Combined with V Flow technology ultrasound, carotid stenosis could be evaluated more comprehensively with the visual quantification of the blood velocity, streamline, and the measurement of the WSS value. V Flow has potential for distinguishing severe carotid stenosis, which may help patients make clinical decisions about further treatment plans.

### Limitations

There are several limitations in our study. Limited cases of patients were included. The majority of enrolled patients were males, because a larger proportion of individuals with carotid atherosclerosis were men than women. Ultrasound examinations were all performed by a single radiologist, and future clinical studies need to further explore inter- or intra-observer agreement.

## 5. Conclusions

V Flow imaging technology can detect the hemodynamic changes of carotid stenosis. It may be a potential non-invasive imaging tool for the preoperative assessment of symptomatic carotid stenosis and the degree of carotid stenosis in aging people.

## Figures and Tables

**Table 1:** Summary characteristics of the patients.

	Value (Mean ± SD)/Number (Percentage)
Gender Female Male	17 (19.3%) 71 (80.7%)
Age (years)	67.6 (5.4)
Hypertension	42 (47.7%)
Diabetes	9 (10.2%)
Hyperlipidemia	17 (19.3%)
Clinical symptom Symptomatic Asymptomatic	32 (36.4%) 56 (63.6%)
Stenosis Grade Mild Moderate Severe	44 (50.0%) 15 (17.0%) 29 (33.0%)

**Table 2:** The diagnostic value of conventional ultrasound in carotid stenosis.

Conventional Ultrasound	DSA or CTA		Total
	Severe Stenosis (n = 29)	Mild or Moderate Stenosis (n = 59)	
Severe stenosis	23	2	25
Mild or moderate stenosis	6	57	63

## Data Availability

Data available on request due to restrictions ethical. The data presented in this study are available on request from the corresponding author. The data are not publicly available due to restrictions ethical.

## References

[1] Ooi Y.C., Gonzalez N.R. (2014). Management of Extracranial Carotid Artery Disease. Cardiol. Clin..

[2] Wang H., Naghavi M., Allen C., Barber R.M., Carter A., Casey D.C., Charlson F.J., Chen A.Z., Coates M.M., Coggeshall M. (2016). AGlobal, regional, and national life expectancy, all-cause mortality, and cause-specific mortality for 249 causes of death, 1980–2015: A systematic analysis for the Global Burden of Disease Study 2015. Lancet.

[3] Abbott A.L., Bladin C.F., Levi C.R., Chambers B.R. (2007). What should we do with asymptomatic carotid stenosis?. Int. J. Stroke.

[4] Abbott A.L., Paraskevas K.I., Kakkos S.K., Golledge J., Eckstein H.-H., Diaz-Sandoval L.J., Cao L., Fu Q., Wijeratne T., Leung T.W. (2015). Systematic Review of Guidelines for the Management of Asymptomatic and Symptomatic Carotid Stenosis. Stroke.

[5] Yan Z., Liang Y., Shi J., Cai C., Jiang H., Song A., Qiu C. (2015). Carotid stenosis and cognitive impairment amongst older Chinese adults living in a rural area: A population-based study. Eur. J. Neurol..

[6] Aboyans V., Björck M., Brodmann M., Collet J.P., Czerny M., De Carlo M., Naylor A.R., Roffi M., Tendera M., Vlachopoulos C. (2018). 2017 ESC Guidelines on the Diagnosis and Treatment of Peripheral Arterial Diseases, in collaboration with the European Society for Vascular Surgery (ESVS): Document covering atherosclerotic disease of extracranial carotid and vertebral, mesenteric, renal, upper and lower extremity arter-iesEndorsed by: The European Stroke Organization (ESO)The Task Force for the Diagnosis and Treatment of Peripheral Arterial Diseases of the European Society of Cardiology (ESC) and of the European Society for Vascular Surgery (ESVS). Eur. Heart J..

[7] Zhang R., Zhang Q., Ji A., Lv P., Zhang J., Fu C., Lin J. (2020). Identification of high-risk carotid plaque with MRI-based radiomics and machine learning. Eur. Radiol..

[8] Saba L., Lai L., Lucatelli P., Sanfilippo R., Montisci R., Suri J.S., Faa G. (2018). Association between carotid artery plaque inflammation and brain MRI. J. Neuroradiol..

[9] Deng F., Mu C., Yang L., Yi R., Gu M., Li K. (2021). The Differentiation in Image Post-processing and 3D Reconstruction During Evalu-ation of Carotid Plaques from MR and CT Data Sources. Front. Physiol..

[10] Jensen S.K., Eesa M., Walker A., Archer D.P., Davis M.J. (2021). Plaque Characteristics on CT Angiography Do Not Improve the Ability to Predict Hemodynamic Instability During and After Carotid Angioplasty and Stenting. J. Neurosurg. Anesthesiol..

[11] Kim E.S., Zierler R.E. (2020). Variation in Ultrasound Diagnostic Thresholds for Carotid Stenosis in the United States. Circulation.

[12] Chappell F.M., Wardlaw J.M., Young G.R., Gillard J.H., Roditi G.H., Yip B., Pell J., Rothwell P.M., Brown M.M., Gough M.J. (2009). Carotid Artery Stenosis: Accuracy of Noninvasive Tests—Individual Patient Data Meta-Analysis. Radiology.

[13] Rothwell P.M., Mehta Z., Howard S.C., Gutnikov S.A., Warlow C.P. (2005). Treating individuals 3: From subgroups to individuals: General principles and the example of carotid endarterectomy. Lancet.

[14] Goddi A., Bortolotto C., Raciti M.V., Fiorina I., Aiani L., Magistretti G., Sacchi A., Tinelli C., Calliada F. (2018). High-Frame Rate Vector Flow Imaging of the Carotid Bifurcation in Healthy Adults: Comparison with Color Doppler Imaging. J. Ultrasound Med..

[15] Qiu Y., Dong Y., Mao F., Zhang Q., Yang D., Chen K., Shi S., Zuo D., Tian X., Yu L. (2021). High-Frame Rate Vector Flow Imaging Technique: Initial Application in Evaluating the Hemodynamic Changes of Carotid Stenosis Caused by Atherosclerosis. Front. Cardiovasc. Med..

[16] Goddi A., Bortolotto C., Fiorina I., Raciti M.V., Fanizza M., Turpini E., Boffelli G., Calliada F. (2017). High-frame rate vector flow imaging of the carotid bifurcation. Insights Imaging.

[17] Goddi A., Fanizza M., Bortolotto C., Raciti M.V., Fiorina I., He X., Du Y., Calliada F. (2017). Vector flow imaging techniques: An innovative ultraso-nographic technique for the study of blood flow. J. Clin. Ultrasound.

[18] Qiu Y., Yang D., Zhang Q., Chen K., Dong Y., Wang W.-P. (2020). V Flow technology in measurement of wall shear stress of common carotid arteries in healthy adults: Feasibility and normal values. Clin. Hemorheol. Microcirc..

[19] Zhang X., Yao Z.-Q., Karuna T., He X.-Y., Wang X.-M., Li X.-F., Liu W.-C., Li R., Guo S.-Q., Chen Y.-C. (2018). The role of wall shear stress in the parent artery as an independent variable in the formation status of anterior communicating artery aneurysms. Eur. Radiol..

[20] Du Y., Goddi A., Bortolotto C., Shen Y., Dell’Era A., Calliada F., Zhu L. (2020). Wall Shear Stress Measurements Based on Ultrasound Vector Flow Imaging: Theoretical Studies and Clinical Examples. J. Ultrasound Med..

[21] Azizyan A., Sanossian N., Mogensen M., Liebeskind D. (2010). Fluid-Attenuated Inversion Recovery Vascular Hyperintensities: An Important Imaging Marker for Cerebrovascular Disease. Am. J. Neuroradiol..

[22] Zhao X., Li R., Hippe D.S., Hatsukami T.S., Yuan C., Investigators C.-I. (2017). Chinese Atherosclerosis Risk Evaluation (CARE II) study: A novel cross-sectional, multicentre study of the prevalence of high-risk atherosclerotic carotid plaque in Chinese patients with ischaemic cerebrovascular events-design and rationale. Stroke Vasc. Neurol..

[23] Howard D.P., Van Lammeren G.W., Rothwell P.M., Redgrave J.N., Moll F.L., de Vries J.P.P., De Kleijn D.P., Den Ruijter H.M., De Borst G.J., Pasterkamp G. (2015). Symptomatic carotid atherosclerotic disease: Correlations between plaque composition and ipsilateral stroke risk. Stroke.

[24] (1991). North American Symptomatic Carotid Endarterectomy Trial Collaborators. Beneficial effect of carotid endarterectomy in symptomatic patients with high-grade carotid stenosis. N. Engl. J. Med..

[25] Grant E.G., Benson C.B., Moneta G.L., Alexandrov A.V., Baker J.D., Bluth E.I., Carroll B.A., Eliasziw M., Gocke J., Hertzberg B.S. (2003). Carotid Artery Stenosis: Gray-Scale and Doppler US Diagnosis—Society of Radiologists in Ultrasound Consensus Conference. Radiology.

[26] Hua Y., Jia L., Xing Y., Hui P., Meng X., Yu D., Pan X., Fang Y., Song B., Wu C. (2019). Distribution Pattern of Atherosclerotic Stenosis in Chinese Patients with Stroke: A Multicenter Registry Study. Aging Dis..

[27] Rahman M.S., Woollard K. (2017). Atherosclerosis. Adv Exp. Med. Biol..

[28] Toba T., Otake H., Choi G., Kim H.J., Onishi H., Sugizaki Y., Takeshige R., Nagasawa A., Nagano Y., Tsukiyama Y. (2021). Wall Shear Stress and Plaque Vulnerability: Computational Fluid Dynamics Analysis Derived From cCTA and OCT. JACC Cardiovasc. Imaging.

[29] Hartman E.M.J., De Nisco G., Gijsen F.J.H., Korteland S.-A., van der Steen A.F.W., Daemen J., Wentzel J.J. (2021). The definition of low wall shear stress and its effect on plaque progression estimation in human coronary arteries. Sci. Rep..

[30] Eshtehardi P., Brown A.J., Bhargava A., Costopoulos C., Hung O.Y., Corban M.T., Hosseini H., Gogas B.D., Giddens D.P., Samady H. (2017). High wall shear stress and high-risk plaque: An emerging concept. Int. J. Cardiovasc. Imaging.

[31] Fukumoto Y., Hiro T., Fujii T., Hashimoto G., Fujimura T., Yamada J., Okamura T., Matsuzaki M. (2008). Localized Elevation of Shear Stress Is Related to Coronary Plaque Rupture: A 3-Dimensional Intravascular Ultrasound Study with In-Vivo Color Mapping of Shear Stress Distribution. J. Am. Coll. Cardiol..

[32] Tuenter A., Selwaness M., Lorza A.A., Schuurbiers J., Speelman L., Cibis M., van der Lugt A., de Bruijne M., van der Steen A., Franco O. (2016). High shear stress relates to intraplaque haemorrhage in asymptomatic carotid plaques. Atherosclerosis.

[33] Du Y., Shen Y., Yiu B.Y., Alfred C.H., Zhu L. High Frame rate vector flow imaging: Development as a new diagnostic mode on a clinical scanner. Proceedings of the IEEE International Ultrasonics Symposium.

[34] He L., Cai Y., Feng Y., Wang W., Feng T., Shen E., Yang S. (2022). Utility of vector flow mapping technology in quantitative as-sessment of carotid wall shear stress in hypertensive patients: A preliminary study. Front. Cardiovasc. Med..

[35] Evans D.H., Jensen J.A., Nielsen M.B. (2011). Ultrasonic colour Doppler imaging. Interface Focus.

